# Exosomal microRNA let-7c-5p enhances cell malignant characteristics by inhibiting TAGLN in oral cancer

**DOI:** 10.32604/or.2024.048191

**Published:** 2024-09-18

**Authors:** YI LI, TIANYI WANG, HAORAN DING, SHIYONG ZHUANG, XIAOBO DAI, BING YAN

**Affiliations:** 1 State Key Laboratory of Oral Diseases & National Center for Stomatology & National Clinical Research Center for Oral Diseases & Department of Head and Neck Oncology Surgery, West China Hospital of Stomatology, Sichuan University, Chengdu, 610041, China; 2 Frontier Innovation Center for Dental Medicine Plus, Chengdu, 610041, China

**Keywords:** Oral cancer, Exosomal microRNA, let-7c-5p, Tumorigenesis biomarkers, Gene expression profiling, TAGLN

## Abstract

**Background:**

Oral cancer, a malignancy that is prevalent worldwide, is often diagnosed at an advanced stage. MicroRNAs (miRNAs) in circulating exosomes have emerged as promising cancer biomarkers. The role of miRNA let-7c-5p in oral cancer remains underexplored, and its potential involvement in tumorigenesis warrants comprehensive investigation.

**Methods:**

Serum samples from 30 patients with oral cancer and 20 healthy controls were used to isolate exosomes and quantify their RNA content. Isolation of the exosomes was confirmed through transmission electron microscopy. Quantitative PCR was used to assess the miRNA profiles. The effects of let-7c-5p and TAGLN overexpression on oral cancer cell viability, migration, and invasion were analyzed via CCK-8 and Transwell assays. Moreover, we conducted mRNA sequencing of exosomal RNA from exosomes overexpressing let-7c-5p to delineate the gene expression profile and identify potential let-7c-5p target genes.

**Results:**

let-7c-5p was upregulated in serum-derived exosomes of patients with oral cancer. Overexpression of let-7c-5p in the TCA8113 and CAL-27 cell lines enhanced their proliferative, migratory, and invasive capacities, and overexpression of let-7c-5p cell-derived exosomes promoted oral cancer cell invasiveness. Exosomal mRNA sequencing revealed 2,551 differentially expressed genes between control cell-derived exosomes and overexpressed let-7c-5p cell-derived exosomes. We further identified TAGLN as a direct target of let-7c-5p, which has been implicated in modulating the oncogenic potential of oral cancer cells. Overexpression of TAGLN reverses the promoting role of let-7c-5p on oral cancer cells.

**Conclusion:**

Our findings highlight the role of exosomal let-7c-5p in enhancing oral cancer cell aggressiveness by downregulating TAGLN expression, highlighting its potential as a diagnostic and therapeutic strategy.

## Introduction

Oral cancer is a significant global health issue that primarily manifests as oral squamous cell carcinoma (OSCC, accounting for 90% of all cases), with an alarming trend of increased incidence and mortality rates [1]. Oral cancer is known for its aggressive progression, early dissemination, and high propensity for loco-regional recurrence [2]. Despite advancements in surgical and chemoradiotherapy strategies, the five-year survival rate for patients with oral cancer remains very poor, at less than 40% [3], with frequent late-stage detection [4]. Therefore, early diagnostic biomarkers and innovative therapeutic targets are required [5]. The etiological landscape of oral cancer is multifaceted and involves a confluence of genetic, epigenetic, and environmental factors, with tobacco and alcohol consumption being well-established risk contributors [6,7]. The intricacies of the molecular pathology of oral cancer remain largely unknown; thus there is a critical need to understand the underlying molecular mechanisms that drive its development and progression.

There is increasing evidence that extracellular vesicles, particularly exosomes, are significant players in cancer biology, serving as vehicles for intercellular communication and modulating numerous physiological and pathological processes, such as tumorigenesis, the tumor microenvironment, and angiogenesis [8,9]. These nano-sized vesicles encapsulate a diverse array of biomolecules, including proteins, lipids, and a rich repertoire of RNA species, among which microRNAs (miRNAs) have garnered considerable interest [7,10]. miRNAs, in their role as post-transcriptional regulators of gene expression, can influence a plethora of cellular pathways and have been implicated in the pathogenesis of various cancers, including oral carcinoma [11]. Exosomal miRNAs, owing to their stability in bodily fluids and their ability to reflect the disease state, present a promising frontier for noninvasive biomarker discovery [7]; one example of these developments is the report that exosomal miR-1307-5p is indicative of aggressive disease and unfavorable outcomes in patients with OSCC [12]. miRNA-4497 has been demonstrated to be a potential biomarker for metastasis and prognosis in non-small-cell lung cancer [13]. Overexpression of miR21 in epithelial ovarian cancer promotes tumor cell proliferation, migration, and invasion while inhibiting apoptosis [14]. Research focused on exosomal miRNAs holds great promise as a source of new insights into tumor biology and, consequently, the identification of novel avenues for diagnosis and therapy.

Within this miRNA landscape, let-7c-5p has emerged as a molecule of interest because of its documented prognostic functions in multiple cancer contexts [15,16]. The role of let-7c-5p in regulating cell proliferation, apoptosis, and metastasis has been established in liver cancer, clear cell renal cell carcinoma, and lung adenocarcinoma [17–19]. Preliminary studies have suggested that exosomal let-7c-5p is a pivotal factor in modulating the tumor microenvironment, influencing the behavior of both cancerous and surrounding stromal cells [20]. Jayaseelan et al. reported that the exosomal miRNA let-7c-5p is differentially expressed and significantly affects the survival rates of patients with head and neck squamous cell carcinomas [21]. However, the role of let-7c-5p in oral cancer pathology remains unclear; to date, there is only one published report of this interaction: by binding to let-7c-5p, the lncRNA MIR4713HG exacerbates the malignant behavior of oral tongue squamous cell carcinoma [22]. Moreover, let-7c-5p has been shown to exhibit reversed effects on tumors in different cancer types. For example, let-7c-5p is highly expressed in liver cancer and inhibits tumor growth [17]; however, it is expressed at low levels in lung cancer and promotes tumor invasion in clear-cell renal cell carcinoma [18]. Given the dual nature of let-7c-5p as a potential suppressor or enhancer of tumor progression in different cancer types, there is a compelling need to elucidate its specific role in oral cancer.

In the present study, we investigated the role of the serum-derived exosomal miRNA let-7c-5p in oral cancer. Through the validation of target genes, we shed light on the oncogenic mechanisms potentially governed by let-7c-5p. With this study we not only seek to advance the understanding of exosomal miRNAs in oral cancer pathogenesis but also pave the way for developing innovative therapeutic strategies, leveraging the nuanced roles of miRNAs in tumor biology.

## Materials and Methods

### Samples and plasma isolation

Peripheral blood samples were obtained from patients with oral cancer (n = 30) and healthy individuals (n = 20) from the West China Hospital of Stomatology, Sichuan University; the blood was collected under fasting conditions (pre-breakfast) and drawn into EDTA-containing blood collection tubes. Plasma fractions were immediately separated by centrifugation at 3,000 g for 15 min at 4°C and kept in RNAse-free EP tubes at −80°C. This study was approved by the West China Hospital of Stomatology, Sichuan University (WCHSIRB-D-2023-438), and all patients signed an informed consent form prior to sample collection.

### Extraction of exosomes and identification via transmission electron microscopy (TEM)

Plasma exosomes were extracted through ultracentrifugation and identified via TEM as described previously [23].

### Nanoparticle tracking analysis (NTA)

For nanoparticle tracking analysis, a 10-μL volume of exosomes was diluted by adding it to 30 μL of PBS. Size distribution was analyzed using a flow analyzer (NanoAnalyzer #N30E, Fujian, China).

### Fluorescent labeling and nanoflow detection of exosomal samples

Diluted exosomes were incubated with fluorescently labeled antibodies (20 μL) of CD9 (#555371, BD, NJ, USA), CD63 (#556019, BD), or IgG (#400108, BioLegend, CA, USA) for 30 min in the dark. After the addition of 1 mL of PBS, the exosomes were centrifuged twice at 110,000 g for 70 min and analyzed using a flow analyzer (NanoAnalyzer #N30E, Fujian, China).

### Exosomes tracking

A PKH26 kit (#UR53202, Umibio, Shanghai, China) was used for the uptake studies. Briefly, 1 μL PKH26 dye was added to 9 μL Diluent C, which was then used to incubate exosomes for 10 min in the dark. The labeled exosomes were collected by centrifugation at 100,000 g for 17 min. Nuclei were stained with DAPI for 10 min. The resulting images were acquired using a laser-scanning confocal microscope (Olympus, Tokyo, Japan).

### Exosomal RNA extraction and quantitation

Exosomal RNA was isolated from the exosomes using the miRNeasy Mini Kit (#217004, Qiagen, Hilden, Germany). The RNA concentration was determined using a Quantus Fluorometer (Promega, Madison, WI, USA). The extracted RNA was stored at −80°C until further use.

### Cell culture and transfection

The human oral cancer cell lines TCA8113 and CAL-27 were cultured in DMEM (#SH30243.1, Gibco, Waltham, MA, USA) with 1% penicillin streptomycin mixture (#MP 1670249-M, Gibco,) and 10% fetal bovine serum (#10099-141, Gibco). Hoechst DNA staining showed no mycoplasma infection. Oe-let-7c-5p TCA8113 cells were transfected with oe-TAGLN vector.

### Cell viability assay using cell counting kit 8 (CCK-8)

Cells (5,000 cells/well) were plated in 96-well plates and incubated for 48 h, after which post-treatment, CCK-8 reagent (10 μL) was added, and the plates were incubated for an additional 2 h. Absorbance was measured at 450 nm using a microplate reader (Bio-Rad Laboratories, CA, USA).

### Transwell assays for cell migration and invasion

Transwell inserts (Corning, NY, USA) were used. For the migration assay, 2 × 10^5^ cells in serum-free medium were seeded in the upper chamber, and the medium was placed in the lower chamber. After a 24-h incubation period, the cells that migrated to the lower surface were fixed with 4% paraformaldehyde, stained with 0.1% crystal violet, and counted under a light microscope (Olympus, Tokyo, Japan).

A wound-healing assay was also used to evaluate cell migration. Cells were plated in 6-well plates (7 × 10^5^ cells/well), scratched using a sterilized pipette, and incubated in serum-free DMEM for 24 h.

For the invasion assay, Transwell inserts were coated with Matrigel (#356234, BD, CA, USA) diluted in a serum-free medium to form a matrix barrier. Cell preparation, processing, fixation, staining, and quantitation were similar to those in the migration assay.

### mRNA sequencing and data analysis

mRNA was selectively isolated from the previously extracted exosomal RNA using the Dynabeads mRNA DIRECT Kit (#61011, Invitrogen, Waltham, MA, USA), which employs magnetic beads for the direct capture of polyadenylated mRNA molecules. Following isolation, sequencing libraries were constructed using the TruSeq Stranded mRNA Library Prep Kit (Illumina, San Diego, CA, USA), designed to preserve strand orientation and ensure comprehensive coverage of the transcriptome. The prepared libraries were sequenced on an advanced Illumina sequencing platform to generate high-throughput sequencing data.

After sequencing, raw data quality control checks were performed systematically using the FastQC software (Babraham Bioinformatics, Cambridge, UK). Subsequent to quality verification, differential gene expression analysis was undertaken with DESeq2 (Bioconductor, https://www.bioconductor.org/), adhering to a selection criterion based on statistical significance *p* < 0.05 and |Log_2_FC| ≥ 1.

Enrichment analyses were performed using the ClusterProfiler package in R. This comprehensive analysis encompassed Gene Ontology (GO), Kyoto Encyclopedia of Genes and Genomes (KEGG) pathways, Reactome pathways, and Disease Ontology (DO).

### Identification of target genes for let-7c-5p

Following mRNA sequencing and subsequent enrichment analyses, we validated the target genes modulated by hsa-let-7c-5p. Initially, we filtered downregulated DEGs from the mRNA-seq dataset. Using the multiMiR R package, we retrieved a comprehensive list of predicted and experimentally validated targets of hsa-let-7c-5p. This list was then intersected with our mRNA-seq-derived downregulated DEGs to isolate the effective targets within our experimental context. For further validation and to ensure the oral cancer specificity of these targets, we cross-referenced the identified genes using the Oral Cancer Gene Database (OCDB, Version II, https://www.actrec.gov.in/OCDB/). The final list of target genes for hsa-let-7c-5p was refined by retaining only the genes found in the OCDB.

### Reverse transcription quantitative polymerase chain reaction (RT-qPCR)

Total RNA was extracted from cell or exosome samples using the miRNeasy Mimi kit (#217004, Qiagen, Hilden, Gemany). The cDNA synthesis kit should be: RevertAid First Strand cDNA Synthesis Kit (#00597742, Invitrogen, Waltham, MA, USA) C1000 Touch Thermal Cycler (#CFX96, Bio-Rad Laboratories, California, USA). qRT-PCR was performed using the SYBR Green Supermix on a CFX Connect Real-Time PCR Detection System (Bio-Rad Laboratories). Primers used are listed in Suppl. Table S1. Relative gene expression was calculated using the 2^-ΔΔCt^ method, normalizing to internal reference genes U6/GAPDH or external reference Cel-miR-39 [24].

### Western blotting

Western blotting was performed as previously described [25]. Primary antibodies included Calnexin (1:1,000; #ab22595, Abcam, Cambridge, UK), anti-HOXA1 (1:1,000; #ab230513, Abcam), anti-TAGLN (1:1,000; #ab213273, Abcam), and anti-β-actin (1:3,000; #ab8227, Abcam).

### Luciferase reporter assay

The 3′UTR region of TAGLN containing the predicted let-7c-5p binding site was cloned downstream of the luciferase gene in the psiCHECK-2 vector (Promega). Cells were co-transfected with the reporter construct and the let-7c-5p mimic using Lipofectamine 3000. Luciferase activity was measured using a Dual-Luciferase Reporter Assay System (Promega).

### Statistical analysis

Statistical analyses were performed using GraphPad Prism software (version 7.0, San Diego, California, USA). Differences between groups were evaluated using the Student’s *t*-test. *p* < 0.05 was considered statistically significant. All experiments were conducted at least in triplicate.

## Results

### Serum-derived exosomal miRNA let-7c-5p is upregulated in patients with oral cancer

**Figure 1:**
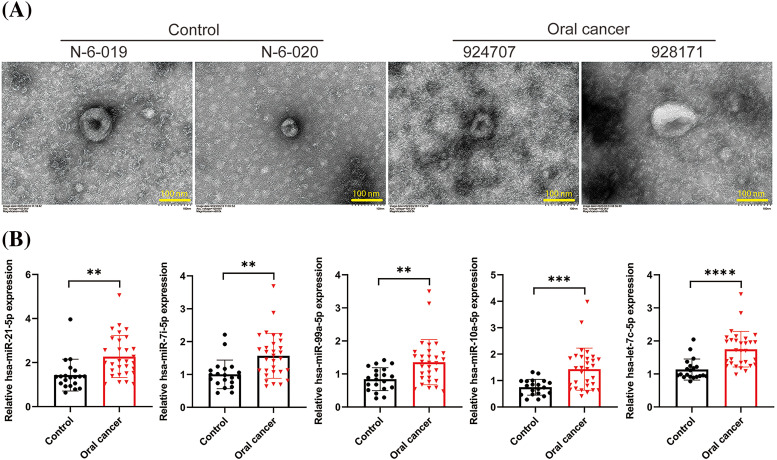
Exosomal miRNA expression in oral cancer and control samples. (A) Transmission electron microscopy (TEM) images of exosomes isolated from the serum of healthy controls (N-6-019, N-6-020) and oral cancer patients (924707, 928171). Scale bars = 100 nm. (B) Quantitative analysis of exosomal miRNA levels, including miR-21-5p, miR-17-5p, miR-93-5p, and let-7c-5p in control and oral cancer samples. Data are presented as mean ± SD; **p < 0.01, ***p < 0.001, ****p < 0.0001.

To investigate the differences in exosomal RNA from the oral cavity between individuals with oral cancer and healthy subjects, we collected serum samples from 30 patients diagnosed with oral cancer and 20 healthy controls. Exosomes were isolated, and the RNA content was quantified. Representative TEM images of exosomes from two healthy subjects (N-6-019 and N-6-020) and two patients with oral cancer (924707 and 928171) are shown in Fig. 1A. These images exhibited a classic cup-shaped morphology, confirming successful exosome extraction. The concentrations of exosomal RNA across the samples are presented in Table 1. Furthermore, we quantified five miRNAs within the extracted exosomes: hsa-miR-21-5p, hsa-let-7i-5p, hsa-miR-99a-5p, hsa-miR-10a-5p, and hsa-let-7c-5p, which were the most significantly differentially expressed in the normal and cancer samples. Notably, all these miRNAs were significantly upregulated in the serum exosomes from the oral cancer patients (*p* < 0.01), with let-7c-5p demonstrating the most pronounced elevation (*p* < 0.0001; Fig. 1B). Given its marked upregulation, let-7c-5p was selected for subsequent experiments.

**Table 1:** RNA concentration extracted from exosomes in 50 samples

Group	Samples	RNA concentration (ng/µL)	Total RNA (ng)
Control	N-6-020	0.74	18.5
	N-6-019	1.8	45
	N-6-018	0.8	20
	N-6-017	0.86	21.5
	N-6-016	1.8	45
	N-6-015	0.62	15.5
	N-6-014	0.27	6.75
	N-6-013	0.28	7
	N-6-012	0.2	5
	N-6-011	0.22	5.5
	N-6-010	0.22	5.5
	N-6-009	0.23	5.75
	N-6-008	0.19	4.75
	N-6-007	0.26	6.5
	N-6-006	0.2	5
	N-6-005	0.29	7.25
	N-6-004	0.24	6
	N-6-003	0.33	8.25
	N-6-002	0.15	3.75
	N-6-001	0.32	8
Oral cancer	928171	0.43	10.75
	924707	0.53	13.25
	929122	0.23	5.75
	B-6-001	0.25	6.25
	B-5-005	0.29	7.25
	B-5-001	0.3	7.5
	B-5-002	0.78	19.5
	B-4-007	0.27	6.75
	B-4-006	0.43	10.75
	B-4-005	0.2	5
	B-4-004	0.24	6
	B-4-003	0.26	6.5
	B-4-001	0.22	5.5
	BC0031	0.44	11
	BC0032	0.33	8.25
	BC0030	0.26	6.5
	BC0034	0.43	10.75
	BC0033	0.43	10.75
	BC0028	0.29	7.25
	BC0029	0.49	12.25
	BC0027	0.41	10.25
	BC0025	0.3	7.5
	BC0026	0.21	5.25
	BC0024	0.25	6.25
	B-4-002	0.14	3.5
	BC0019	0.28	7
	BC0020	0.37	9.25
	BC0021	0.31	7.75
	BC0023	1.1	27.5
	BC0022	0.36	9

### let-7c-5p promotes proliferation, migration, and invasion of oral cancer cell lines

**Figure 2:**
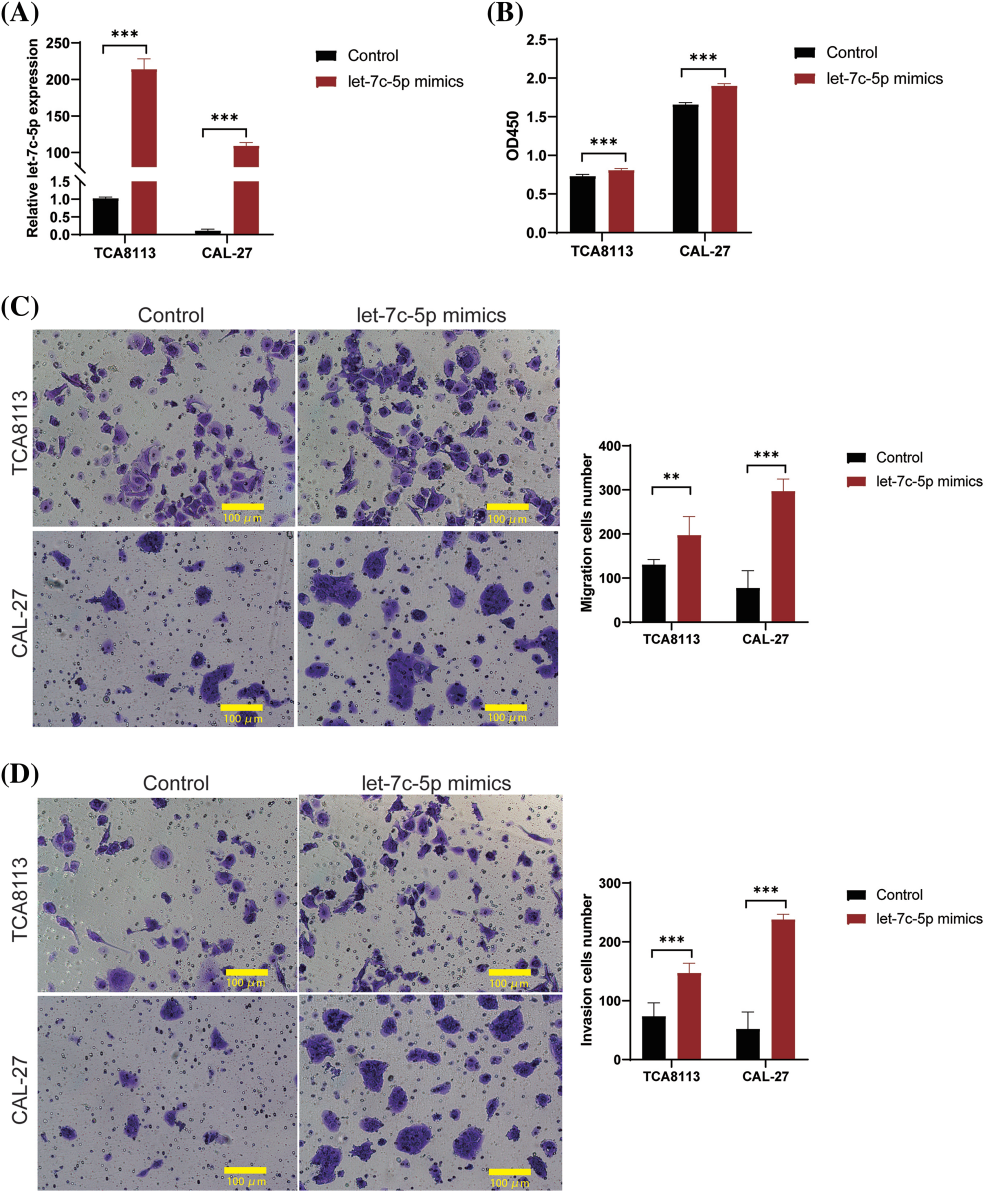
let-7c-5p promotes cell proliferation, migration, and invasion in oral cancer cell lines. (A) Expression levels of let-7c-5p are determined in TCA8113 and CAL-27 cell lines using qPCR. (B) Cell proliferation of TCA8113 and CAL-27 cells is assessed using the CCK-8 assay. (C) Transwell migration assays reveal a marked reduction in cell migration in TCA8113 and CAL-27 cell lines; magnification = 100×; scale bar = 100 µm. (D) Transwell invasion assays demonstrate a significant decrease in invasive cell numbers in both TCA8113 and CAL-27 cell lines compared to control cells; magnification = 100×; scale bar = 100 µm. Oral cancer cell lines TCA8113 and CAL-27 are treated with let-7c-5p mimics. Results are presented as mean ± SD; **p < 0.01, ***p < 0.001.

The role of let-7c-5p in oral cancer has also been previously investigated. We performed functional assays using TCA8113 and CAL-27 cell lines. Initial quantification by qPCR revealed the absence of endogenous let-7c-5p in both cell lines, as indicated in Suppl. Table S2. Subsequently, the cells were treated with let-7c-5p mimic to enhance the intracellular concentration of let-7c-5p. Post-transfection qPCR analysis confirmed was significant overexpression of let-7c-5p in TCA8113 and CAL-27 cells (*p* < 0.001; Fig. 2A), suggesting successful transfection. At 48 h post-transfection, an increase in cell viability was observed in both transfected cell lines compared to that in the untransfected controls (*p* < 0.01; Fig. 2B). Furthermore, the extent of migration and invasion of both TCA8113 and CAL-27 cells was significantly augmented after transfection (*p* < 0.01, Figs. 2C, 2D). These results implicate let-7c-5p as a potential contributor to cell viability and aggressive phenotypes in oral cancer cell lines.

### Exosomal overexpression of let-7c-5p enhances the aggressive phenotype in oral cancer cells

**Figure 3:**
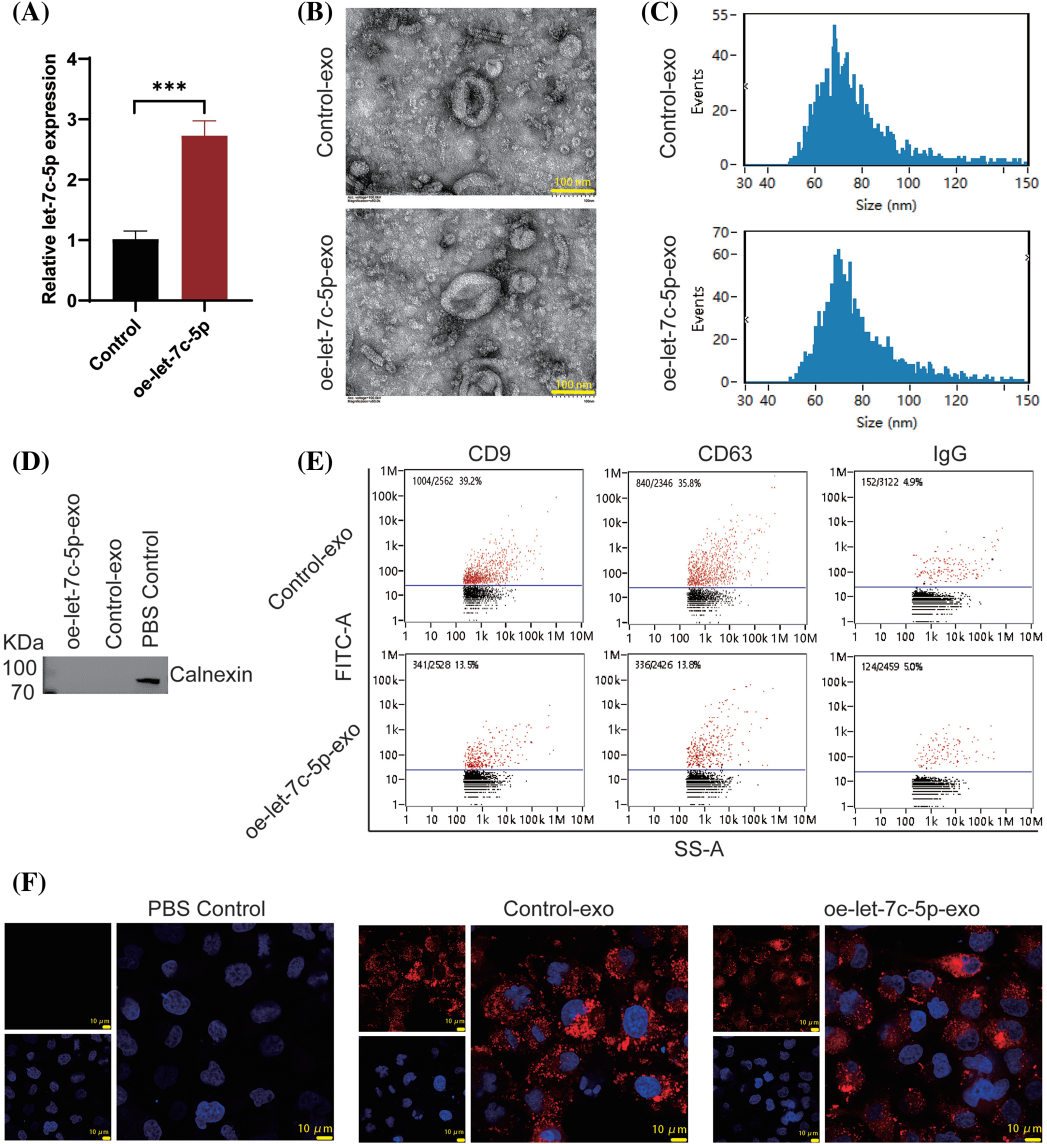
Characterization of Exosomes from Control and oe-let-7c-5p Oral Cancer Cells. (A) let-7c-5p mRNA expression in TCA8113 cells is detected using qPCR; ***p < 0.001. (B) TEM images showing the morphology of exosomes derived from control and let-7c-5p overexpressing cells. Scale bars = 100 nm. (C) Nanoparticle tracking analysis (NTA) displaying the size distribution of exosomes from control and oe-let-7c-5p samples, with peaks indicating the majority of particles within the expected size range for exosomes. (D) Western blotting analysis for the exosome marker Calnexin. (E) Flow cytometry histograms showing the presence of exosomal markers CD9, CD63, and IgG in isolated exosomes. (F) Confocal microscopy images of cells treated with PBS control, control-exo, and oe-let-7c-5p-exo, with exosomes stained in red (PKH26) and nuclei counterstained with DAPI in blue. Scale bars = 10 µm.

To substantiate the role of exosomal let-7c-5p in enhancing the invasive capacity of oral cancer cells, TCA8113 cells were used to overexpress let-7c-5p and exosomes were isolated for functional validation. Following transduction with lentiviral vectors, a marked increase in let-7c-5p expression was detected in TCA8113 cells (*p* < 0.001; Fig. 3A), confirming the efficiency of transfection.

Exosomes were then extracted from treated cells and characterized. TEM analysis yielded images with a characteristic exosomal morphology, displaying the expected size and cup-shaped vesicles (Fig. 3B). The particle size analysis confirmed that the isolated exosomes ranged from 40 to 150 nm in diameter, with exosomes averaging 76.21 nm in the Control-exo group and 77.37 nm in the oe-let-7c-5p-exo group (Fig. 3C). Crucially, the exosome-specific negative marker, calnexin, was not detected in either group, validating the purity of the exosome preparations (Fig. 3D). Conversely, the expression of the exosomal surface markers CD9, CD63, and IgG was confirmed, indicating well-isolated exosome populations (Fig. 3E). Furthermore, both the control-exo and oe-let-7c-5p-exo groups demonstrated robust PKH26 fluorescence, suggesting efficient labeling and tracking of exosomes (Fig. 3F).

**Figure 4:**
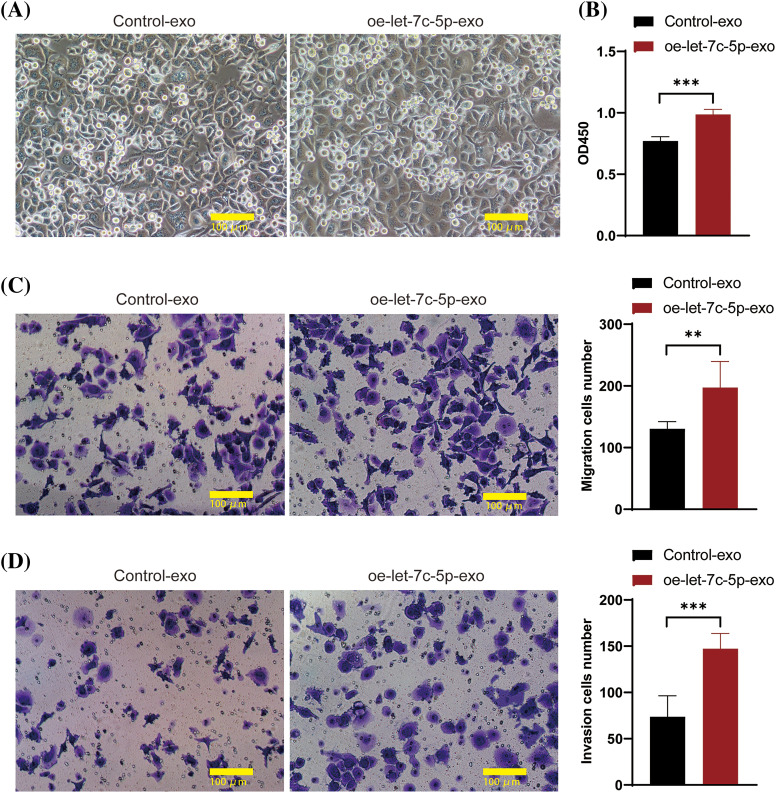
Effects of oe-let-7c-5p-exo on cell proliferation, migration, and invasion. (A) Morphology images of TCA8113 cells after treatment with control-exo and oe-let-7c-5p-exo; magnification = 100×; scale bars = 100 µm. (B) Cell proliferation is determined using CCK-8 in TCA8113 cells. (C and D) Transwell assays for cell migration and invasion in TCA8113 cells; Magnification = 100×; scale bars = 100 µm. Results are presented as mean ± SD; **p < 0.01, ***p < 0.001.

After isolation, TCA8113 cells were treated with harvested exosomes to assess their impact on cellular proliferation, migration, and invasion. The cells maintained a healthy growth state after exosome treatment (Fig. 4A). Comparative analyses revealed that cells treated with oe-let-7c-5p-exo exhibited a significant increase in cell viability, migration, and invasion capacity compared with those treated with control-exo (Figs. 4B–4D). These findings provide compelling evidence that let-7c-5p-enriched exosomes potentiate the malignancy of oral cancer cells.

### Differential gene expression profile in oe-let-7c-5p exosomes in oral cancer

**Figure 5:**
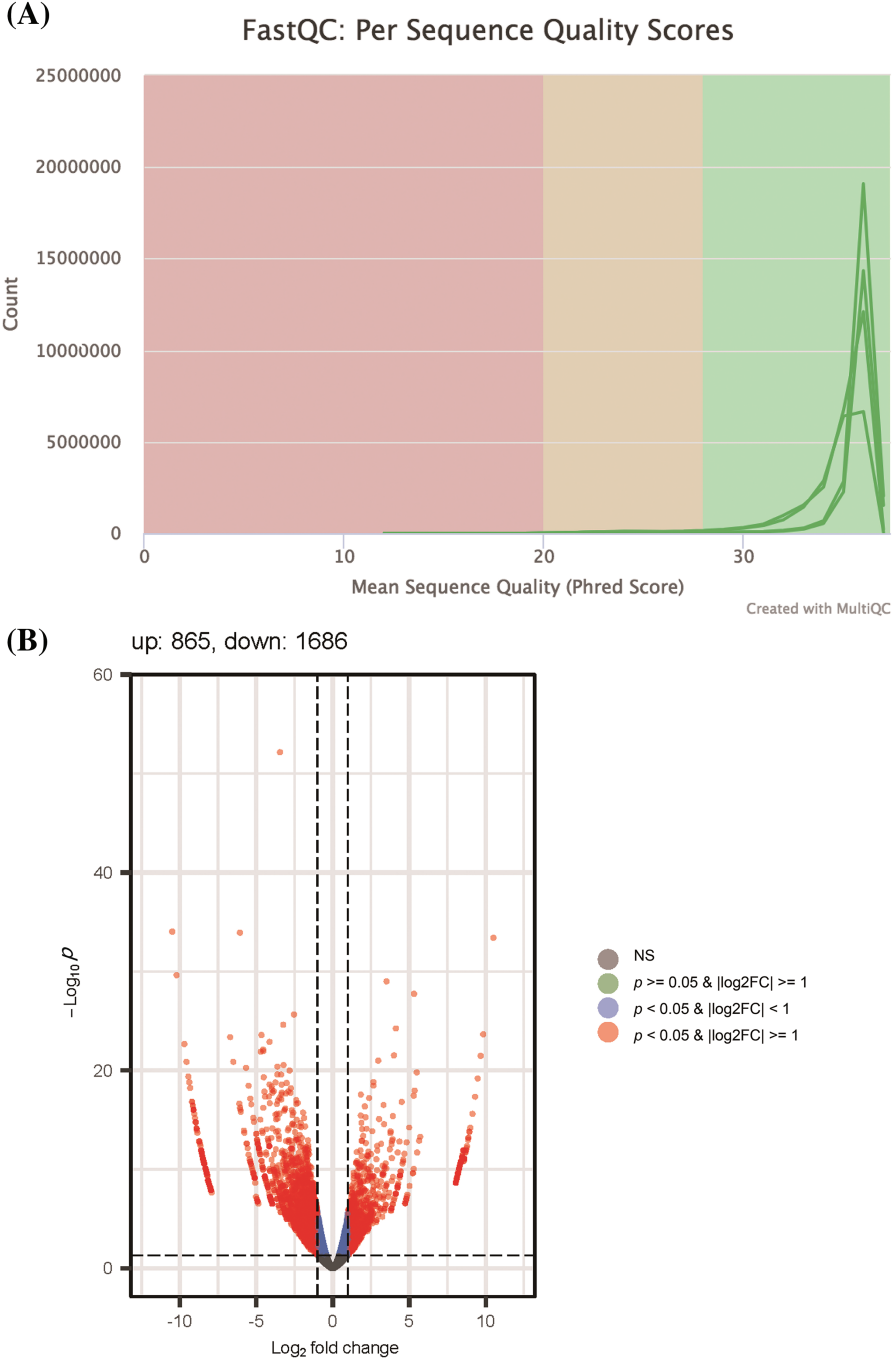
Quality control and differential expression analysis of RNA sequencing data. (A) FastQC per sequence quality scores distribution, illustrating the overall quality of sequencing data across all bases. The graph demonstrates a high mean sequence quality score (Phred Score) with most bases above 28, indicating very good quality. (B) Volcano plot presenting the differential expression between control-exo and oe-let-7c-5p-exo. Each point represents a gene; the red points highlight genes that are significantly differentially expressed (*p*-value < 0.05 & |Log_2_FC| > 1), with the number of upregulated genes (865) and downregulated genes (1686) annotated. The plot shows a higher number of downregulated genes in the dataset. Non-significant genes are depicted in grey.

To determine the potential mechanisms by which let-7c-5p contributes to the malignant phenotype of oral cancer, we performed mRNA sequencing of exosomal RNA isolated from the control and oe-let-7c-5p groups. Sequencing quality assessments confirmed that most bases achieved a quality score above the Q30 threshold, indicating high-fidelity sequence data (Fig. 5A). Subsequent gene quantification and annotation yielded a compendium of 2,551 differentially expressed genes (DEGs), adhering to a selection criterion of a *p*-value < 0.05 and a |Log2FC| ≥ 1. Within this dataset, 865 genes were upregulated and 1,686 were downregulated in the oe-let-7c-5p-exo group (Fig. 5B), as detailed in Suppl. Table S3.

Enrichment analysis integrating the GO, KEGG, Reactome, and DO databases revealed distinct patterns of gene expression alterations (Suppl. Tables S4–S7). The top 20 enriched GO terms predominantly included categories, such as presynapse, passive transmembrane transporter activity, and channel activity (Suppl. Fig. S1A). KEGG pathway enrichment was most pronounced in signaling pathways central to cellular proliferation, such as the Ras, Rap1, and MAPK signaling pathways (Suppl. Fig. S1B). In the Reactome pathway analysis, genes were heavily involved in cancer-related signaling, including PI3K/AKT signaling in cancer, PI3K events in ERBB2 signaling, and constitutive signaling by aberrant PI3K (Suppl. Fig. S2A). DO analysis underscored an unexpected enrichment in psychiatric disorders, particularly mood disorders and bipolar disorder (Suppl. Fig. S2B).

### Identification of let-7c-5p target genes in oral cancer

**Figure 6:**
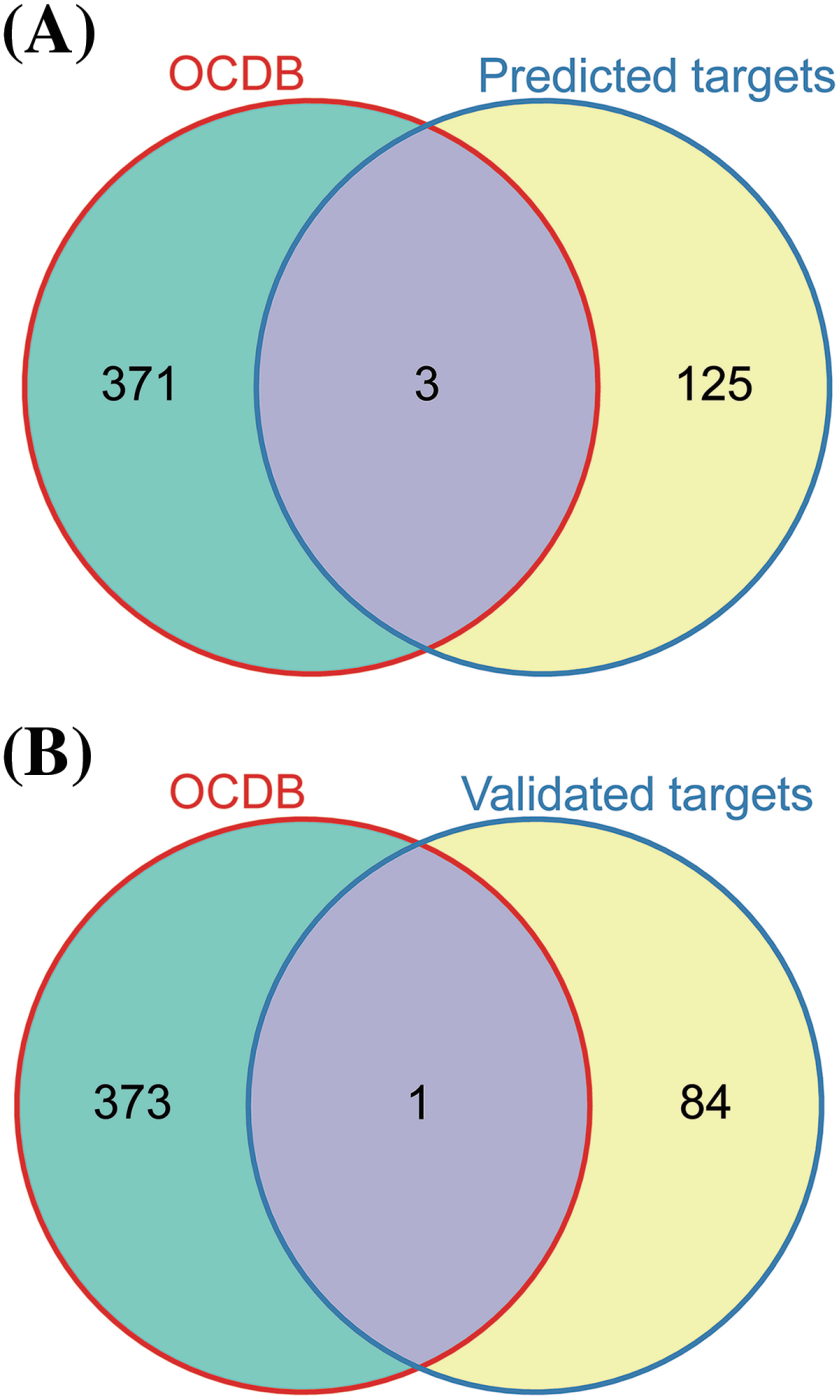
Identification of the let-7c-5p targets in oral cancer. (A) Venn diagram displaying the overlap between genes listed in the OCDB and predicted target genes. (B) Venn diagram showing the overlap between genes in the OCDB and experimentally validated target genes.

Subsequently, we used the multiMiR R package for the comprehensive retrieval of let-7c-5p targets. This package provides a list of genes predicted and previously validated as targets of let-7c-5p. By intersecting the searched and downregulated genes in oe-let-7c-5p-exo, we obtained 128 predicted and 85 experimentally validated target genes. Upon searching the OCDB, we retrieved 374 genes related to oral cancer. The intersection of these oral cancer-related genes with our curated list of predicted and validated let-7c-5p targets resulted in three genes of interest: HOXA1, TAGLN, and TSPAN2 (Figs. 6A, 6B). These genes are potential targets through which let-7c-5p might exert oncogenic effects in oral cancer.

### let-7c-5p targets TAGLN in oral cancer

**Figure 7:**
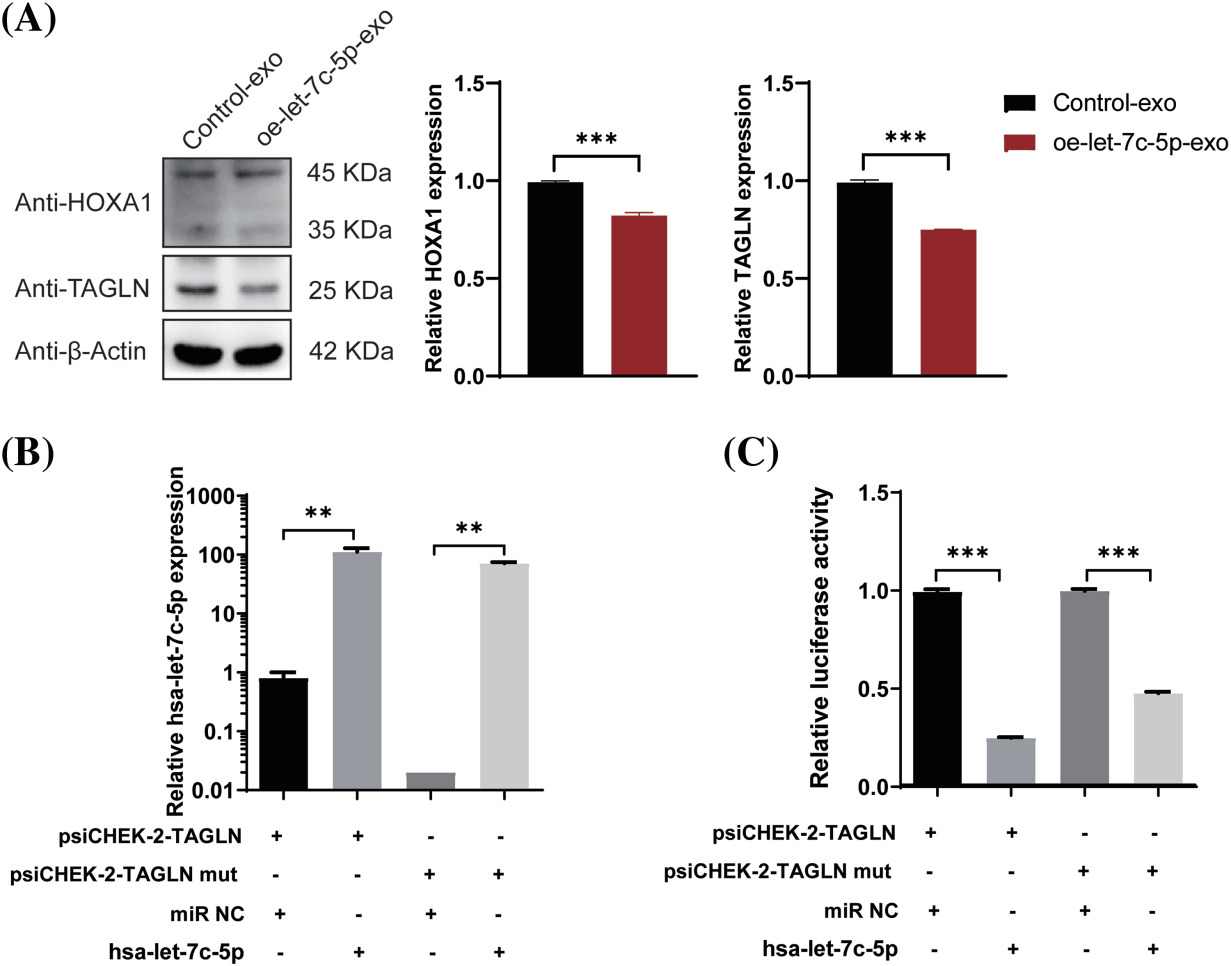
Effects of let-7c-5p on HOXA1 and TAGLN expression and target validation. (A) Western blot analysis and quantification of HOXA1 and TAGLN protein levels in TCA8113 cells treated with control-exo and oe-let-7c-5p-exo. (B) Expression of let-7c-5p after transfection with a let-7c-5p mimics in cells containing the wild-type or mutant TAGLN 3′UTR reporter construct. (C) Relative luciferase activity in cells co-transfected with psiCHECK-2-TAGLN or psiCHECK-2-TAGLN mutant reporter constructs and let-7c-5p mimic. Results are presented as mean ± SD; **p < 0.01, ***p < 0.001.

**Figure 8:**
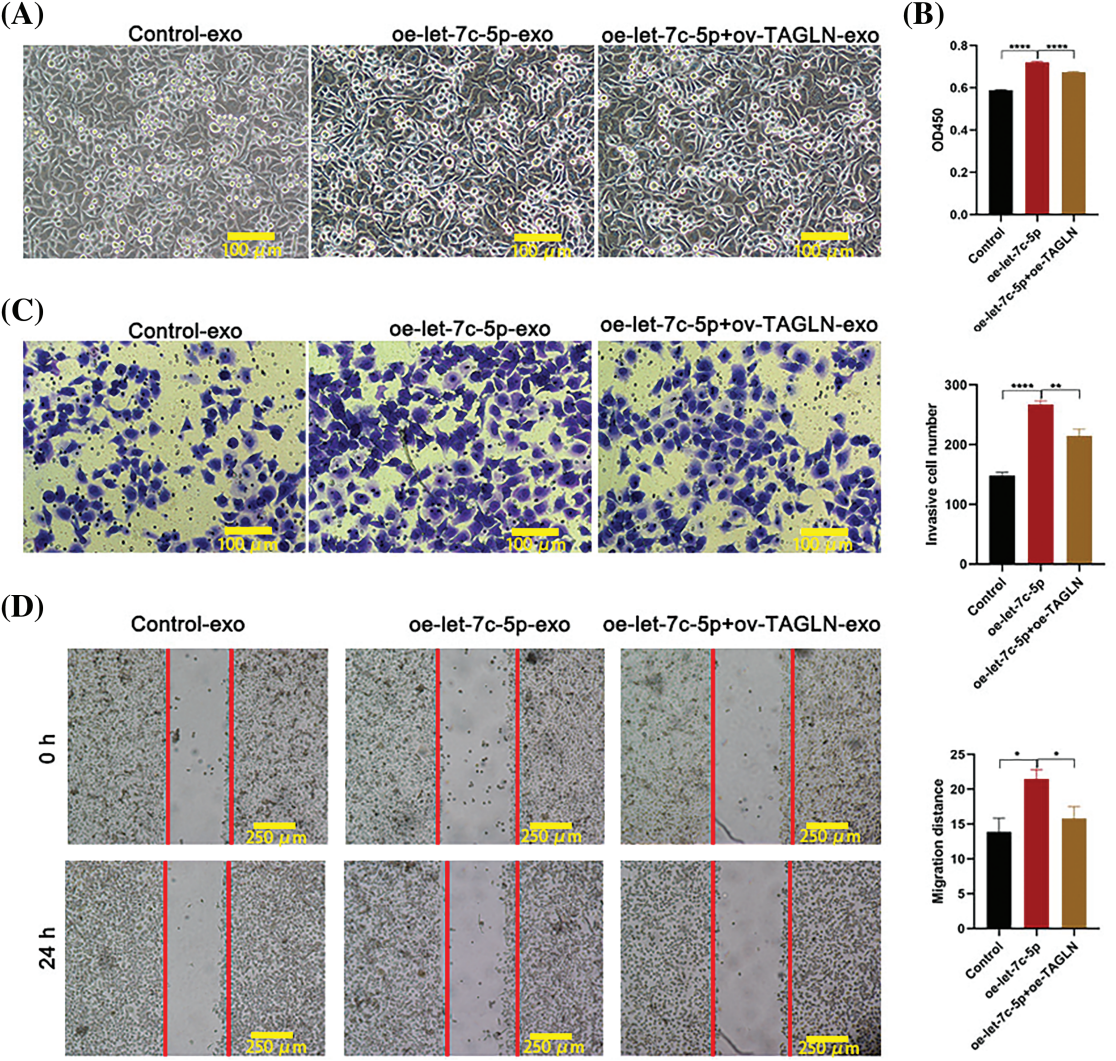
Effects of oe-TAGLN on cell proliferation, migration, and invasion. (A) Morphology images of TCA8113 cells after treatment with control-exo, oe-let-7c-5p-exo, and oe-let-7c-5p+oe-TAGLN-exo; Magnification = 100×; scale bars = 100 µm. (B) Cell proliferation is determined using CCK-8 in TCA8113 cells. (C) Transwell assays for cell invasion in TCA8113 cells. Magnification = 100×; Scale bars = 100 µm. (D) Transwell assays for cell migration in TCA8113 cells; magnification = 100×; scale bars = 250 µm. Results are presented as mean ± SD; *p < 0.05, **p < 0.01, ****p < 0.0001.

Following the identification of the three potential let-7c-5p target genes, their expression levels in TAC8113 cells were examined. Western blot analysis indicated that the presence of oe-let-7c-5p-exo was associated with reduced HOXA1 and TAGLN protein expression in TAC8113 cells (*p* < 0.001; Fig. 7A). Furthermore, a dual-luciferase reporter assay revealed that transfection with let-7c-5p mimics significantly increased let-7c-5p mRNA levels (*p* < 0.01; Fig. 7B). The luciferase assay results demonstrated that let-7c-5p could target the 3′UTR of TAGLN (Fig. 7C). Furthermore, mutations at the predicted binding sites partially reversed the downregulation, suggesting the specificity of the interaction between let-7c-5p and the TAGLN 3′UTR (*p* < 0.001, Fig. 7C). Rescue experiments were performed to further verify the regulatory roles of let-7c-5p and TAGLN. After oe-let-7c-5p + oe-TAGLN treatment, the expression level of TAGLN in the cells was upregulated, demonstrating the success of the cell model (Suppl. Fig. S3). Overexpression of TAGLN inhibited the enhancement of let-7c-5p expression on cell viability, invasion, and migration (Fig. 8).

## Discussion

In this study we presented a comprehensive analysis of the role of the serum-derived exosomal miRNA let-7c-5p in oral cancer, revealing its potential as a disease biomarker and providing insights into its functional role in oral carcinoma progression. By analyzing the serum samples of patients with oral cancer and healthy individuals, we demonstrated that let-7c-5p was upregulated in the exosomes of patients with oral cancer. Moreover, our functional assays suggested that let-7c-5p may contribute to the proliferative, migratory, and invasive behaviors of oral cancer cells, implicating it in the aggressive phenotype associated with malignancy. Differential gene expression profiles further substantiated the influence of let-7c-5p in targeting TAGLN in oral cancer pathobiology. TAGLN rescue can inhibit the aggressive phenotype of let-7c-5pon in cancer cells.

The function of let-7c-5p in cancer has been the subject of extensive research, predominantly highlighting its role as a tumor suppressor in a multitude of cancer types [26]. This is evidenced by studies demonstrating its downregulation in malignancies, such as liver and lung cancer, where its restoration inhibits tumor growth [27,28]. In contrast, our findings illustrated that let-7c-5p is upregulated in serum-derived exosomes of patients with oral cancer, suggesting a divergent, possibly oncogenic role in this specific context. Such a paradoxical role of let-7c-5p may be attributed to the unique microenvironment of oral cancer, both in terms of exposure to environmental carcinogens, such as tobacco and alcohol, and the intrinsic cellular composition that could influence miRNA processing and function [6,29]. Our results, which demonstrated enhanced cell viability and invasiveness in oral cancer cell lines transfected with let-7c-5p mimics, further support the hypothesis that let-7c-5p may function as a tumor suppressor in oral cancer. This finding is corroborated by recent studies that have unveiled the complex, context-dependent roles of miRNAs in cancer, where a single miRNA can act as a tumor suppressor in one tissue type and as an oncogene in another [30]. RGS16 regulated by let-7c-5p promotes glioma progression [31]. The emerging picture is an intricate regulatory network in which miRNAs such as let-7c-5p can have diverse roles depending on the network of gene interactions specific to a cancer type. However, the role of exosomes in the modulation of these effects remains unclear. Exosomes serve as carriers of miRNAs and other oncogenic molecules, facilitating communication between tumor cells and their environment, and potentially aiding the progression and metastasis of cancer [32–34]. Upregulation of let-7c-5p in oral cancer-derived exosomes suggests a functional cargo selectively enriched with components that may enhance tumor aggressiveness. This notion is in line with our experimental observation that exosomes overexpressing let-7c-5p promote a more aggressive phenotype in oral cancer cells.

The interplay between miRNAs and their target genes is the cornerstone of post-transcriptional regulation, influencing a myriad of cellular pathways [35–37]. In the context of oral cancer, our research had identified that TAGLN was downregulated in the presence of oe-let-7c-5p-exo, and overexpression of TAGLN reversed the promoting role of let-7c-5p in cancer cells. TAGLN, a critical component of the actin cytoskeleton, has been implicated in various cellular processes, including migration, invasion, and cellular stability [38]. While traditionally considered a marker of smooth muscle differentiation, recent studies have unveiled the involvement of TAGLN in cancer biology, particularly its upregulation in stromal cells within the tumor microenvironment and its correlation with poor prognosis in several cancers [39]. As an example, Chen et al. showed that TAGLN inhibition stimulates the proliferation, migration, and invasion of Eca-109 cells [40], which contributes to cancer metastasis. The authors also demonstrate that TAGLN enhances ferroptosis, thereby curtailing the aggressive development of esophageal squamous cell carcinoma [40]. Our findings are particularly illuminating as they suggest a novel mechanism wherein let-7c-5p may modulate oral cancer pathophysiology through the downregulation of TAGLN. The qPCR and western blotting analyses demonstrated that let-7c-5p overexpression was inversely correlated with TAGLN levels, implying a potential suppressive role of let-7c-5p on TAGLN expression. Furthermore, direct targeting of TAGLN by let-7c-5p, as confirmed by our dual-luciferase reporter assay, provides a concrete molecular link between this miRNA and its target gene. Such interaction is pivotal for miRNA-mediated gene silencing, typically characterized by the binding of the miRNA to the 3′UTR of target mRNA, leading to its degradation or translational inhibition [41]. The specificity of this interaction is highlighted by our mutation analysis, where alterations in the predicted let-7c-5p binding sites on 3′UTR of TAGLN resulted in the abrogation of repression, confirming the direct regulatory role of let-7c-5p. Our study contributes to this knowledge by suggesting that the let-7c-5p-TAGLN axis is a critical modulator of oral cancer aggressiveness.

Notwithstanding the value of the results reported here, our study had some limitations. First, the regulatory role of exosomal let-7c-5p *in vivo* is unknown, and more thorough validation needs to be conducted. The role of TAGLN in oral cancer has not been fully elucidated, and further experiments are needed. In addition, therapeutic strategies based on let-7c-5p should be developed and validated in clinical settings. Nevertheless, this study validated the promoting role of exosomal let-7c-5p in oral cancer cells by directly targeting and downregulating TAGLN, thus providing a crucial indication for the further application and exploration of relevant agents.

## Conclusion

In conclusion, with this study we propose that let-7c-5p is a potential biomarker for oral cancer; our results evidence the role of let-7c-5p in promoting an aggressive cancer phenotype, possibly through direct targeting and downregulation of TAGLN. These findings advance our understanding of miRNA functions in oral cancer and highlight the intricate regulatory networks orchestrated by miRNAs, such as let-7c-5p. As research in this field progresses, further studies are warranted to validate these findings in larger patient cohorts and to elucidate more let-7c-5p targets, which will be crucial for the development of miRNA-based diagnostic and therapeutic strategies in oral cancer.

## Supplementary Materials

Figure S1 Gene Ontology (GO) and Kyoto Encyclopedia of Genes and Genomes (KEGG) pathway enrichment analysis. A) Bubble chart representing the top 20 GO terms for the differentially expressed genes (DEGs). B) The top 20 KEGG pathways. The GeneRatio is shown along the x-axis the significant pathways are lifted along the y-axis. The bubble size reflects the count of genes in each pathway, and the color indicates the level of statistical significance after *p*-value adjustment.

Figure S2 Reactome Pathway and Disease Ontology (DO) term enrichment analysis. Bubble charts representing A) the top 20 Reactome pathways for the DEGs and B) the enrichment analysis based on the top 5 DO terms. The GeneRatio is plotted on the x-axis, and the diseases associated with the DEGs are listed on the y-axis. The size of each bubble reflects the gene count for each disease term, with the color of the bubble indicating the level of significance after adjustment for multiple tests.

Figure S3 Expression level of TAGLN by quantitative polymerase chain reaction (A) and Western blotting (B). ****p < 0.0001.

## Data Availability

The datasets used and/or analyzed during the current study are available from the corresponding author on reasonable request.

## Supplementary Materials

The supplementary material is available online at https://doi.org/10.32604/or.2024.048191.
