# Supplementary figures and images for: Exosomal microRNA let-7c-5p enhances cell malignant characteristics by inhibiting TAGLN in oral cancer
Source: Oncol Res. 2024 Sep 18;32(10):1623–35. doi: 10.32604/or.2024.048191 (PMC11413824; doi:10.32604/or.2024.048191)

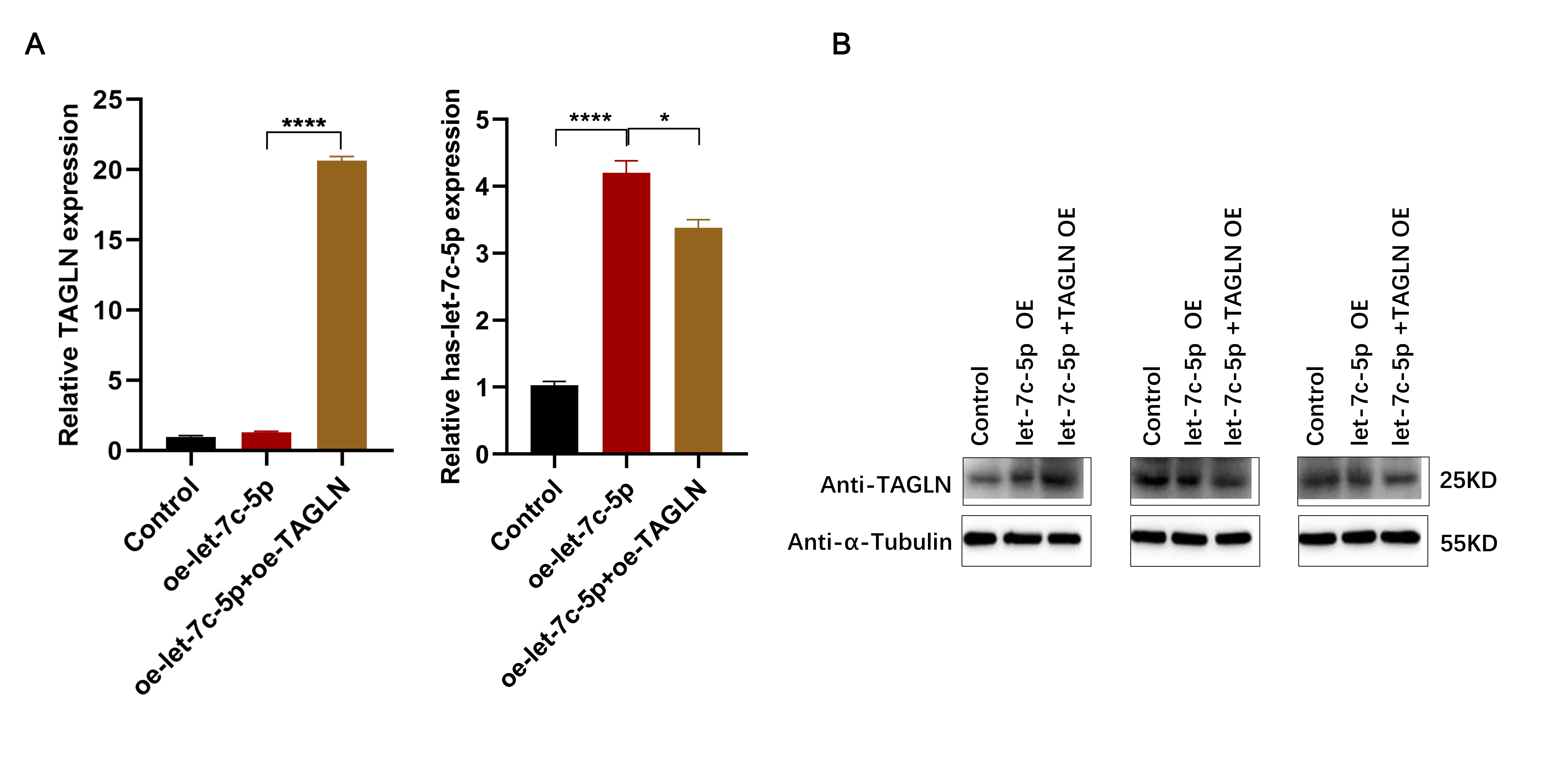

Supplement: Figure S3 [file OncolRes-32-48191-s003.tif]
