# Supplementary material for: Exosomal microRNA let-7c-5p enhances cell malignant characteristics by inhibiting TAGLN in oral cancer
Source: Oncol Res. 2024 Sep 18;32(10):1623–35. doi: 10.32604/or.2024.048191 (PMC11413824; doi:10.32604/or.2024.048191)
Supplement: Supplementary file 4 [file OncolRes-32-48191-s004.docx]

Table S1 Primers in the RT-qPCR analysis

| name | primers |
| --- | --- |
| hsa-miR-21-5p qRT F | TCGGCAGGTAGCTTATCAGACTGA |
| hsa-miR-7156-5p qRT F | TTGTTCTCAAACTGGCT |
| hsa-miR-30d-5p qRT F | TGTAAACATCCCCGAC |
| hsa-let-7i-5p qRT F | TCGGCAGGTGAGGTAGTAGTTTGT |
| hsa-miR-99a-5p qRT F | AACCCGTAGATCCGAT |
| hsa-miR-10a-5p qRT F | TACCCTGTAGATCCGAA |
| hsa-let-7c-5p qRT F | TGAGGTAGTAGGTTGT |
| cel-miR-39 qRT F | AGCCCGTCACCTGGTGTAAATC |
| miR universal qRT R | CAGTGCAGGGTCCGAGGTAT |
| U6 qRT F | CTCGCTTCGGCAGCACA |
| U6 qRT R | AACGCTTCACGAATTTGCGT |
| HOXA1 qRT F | GGGTGTCCTACTCCCACTCA |
| HOXA1 qRT R | GGACCATGGGAGATGAGAGA |
| TAGLN qRT F | AACAGCCTGTACCCTGATGG |
| TAGLN qRT R | CGGTAGTGCCCATCATTCTT |
| TSPAN2 qRT F | CTTGGCTTCAACCTGCTCTT |
| TSPAN2 qRT R | AAGCACACATTGCGACTCC |
| hGAPDH F | CAAGGTCATCCATGACAACTTTG |
| hGAPDH R | GTCCACCACCCTGTTGCTGTAG |
